# Altered regularity and stability of multi-finger force production in individuals with mild cognitive impairment

**DOI:** 10.3389/fnhum.2026.1873097

**Published:** 2026-09-01

**Authors:** Yoon-Seok Choi, Junkyung Song, Jiseop Lee

**Affiliations:** 1 Department of Physical Education, Seoul National University, Seoul, Republic of Korea; 2 Department of Kinesiology, Kyungpook National University, Sangju, Republic of Korea; 3 Division of Geriatrics, Department of Internal Medicine, Yonsei University College of Medicine, Seoul, Republic of Korea; 4 Institute for Innovation in Digital Healthcare, Yonsei University Health System, Seoul, Republic of Korea

**Keywords:** finger coordination, isometric force production, motor dysfunction, non-linear dynamics, uncontrolled manifold hypothesis

## Abstract

While previous studies have identified motor dysfunction in individuals with Mild Cognitive Impairment (MCI), the experimental tasks investigated to date have remained relatively limited. This study compared multi-finger force production in individuals with MCI and healthy controls (HC), employing a task designed to comprehensively reflect the complexities of neural integrity. Participants were instructed to match a target force using the net force of five fingers with real-time visual feedback, while individual finger forces were also measured. The analyses were conducted at two levels: net force level and individual finger force level. In addition, linear and non-linear approaches were utilized to examine spatial and temporal structures. At the net force level, the MCI group exhibited diminished accuracy (RMSE), increased fluctuation (CV), and lower adaptability (SampEn) in force patterns. Furthermore, the MCI group demonstrated significantly lower spatial (ΔV_Z_) and temporal stability (MLE) at individual finger level. These findings suggest that multi-finger tasks may reflect subclinical motor alterations associated with cognitive decline. Consequently, this research offers a valuable evidence-base for the potential development of non-invasive behavioral markers for MCI screening.

## 1 Introduction

Mild cognitive impairment (MCI) is defined as a transitional stage between normal aging and dementia. At the structural level, MCI is recognized as being associated with progressive atrophy and functional alterations in key brain regions (Pa et al., 2009; Tabatabaei-Jafari et al., 2015). Empirical data indicated that while only 1–2% of the general elderly population progresses to dementia annually, this rate escalates to 10–15% among those with MCI (Petersen et al., 2001). Screening for MCI is essential to delay or prevent functional decline; currently, diagnosis primarily utilizes standardized neuropsychological instruments in structured questionnaire formats. However, these questionnaire-based instruments have notable limitations. A primary concern is their susceptibility to demographic variables, such as educational attainment, which can lead to biased scores and potential misclassification (Rossetti et al., 2011; Malek-Ahmadi et al., 2015).

Interestingly, while individuals with MCI do not experience significant motor impairments in daily life, previous studies employing controlled experimental tasks have identified diverse motor deficits (Kluger et al., 1997; Roalf et al., 2018; Mao et al., 2025). This suggests that quantifying these neuromechanical markers could serve as an effective diagnostic approach for MCI (Beauchet et al., 2015; Boripuntakul et al., 2022). The present study employed a multi-finger steady-state force production task which is a suitable tool for assessing visuo-motor integration. Such adjustments rely on closed-loop control mechanisms, in which the prefrontal cortex (PFC) plays a key role in visuo-motor integration (Mandrick et al., 2013; Moro et al., 2016), and previous research has reported potential functional decline or structural alterations within the PFC of individuals with MCI (Han et al., 2012). Furthermore, multi-finger tasks offer distinct advantages, as they require extensive cortical involvement (Binkofski et al., 1999) and motor coordination.

A multi-finger force production task involves a redundant system, meaning that stable motor performance (maintaining a constant net force) is achieved through the compensatory covariation of individual fingers. In contrast, when fingers exhibit positive covariation, it becomes inherently difficult to maintain a constant net force, as fluctuations in one finger are amplified by the others. This flexible organization of redundant elements is recognized as a neurological strategy termed “*synergy*” (Latash et al., 2007). Previous studies suggest that such synergistic actions reflect the functional integrity of neural structure, especially basal ganglia (Park et al., 2012; Park et al., 2014). Because basal ganglia atrophy is one of the symptoms in individuals with MCI (Teipel et al., 2007; Madsen et al., 2010), the current study conducted this analysis to investigate motor performance from this perspective.

Non-linear approaches provide a robust framework to evaluate regularity and stability of systems more comprehensively by characterizing the temporal structure of performance, offering insights that go beyond simple measures of variance magnitude (Stergiou and Decker, 2011; van Emmerik et al., 2016; Strongman and Morrison, 2020). Such approaches may be particularly useful for detecting subtle changes in motor control associated with neurological conditions. Because MCI involves early dysfunction in neural networks contributing to motor planning and control, nonlinear analysis of motor output may provide sensitive markers of altered motor system dynamics. Representative non-linear approaches in biomechanics include sample entropy (SampEn) and the maximum Lyapunov exponent (MLE). Specifically, SampEn serves as an indicator of the irregularity of a system’s output, where lower values reflect more rigid and predictable patterns – often associated with a loss of motor adaptability (Kim et al., 2020). Conversely, the MLE quantifies the rate of divergence in a system’s trajectory, providing a direct measure of dynamic stability. A higher MLE represents lower dynamic stability, indicating how small perturbations affect the system’s consistent performance (Reynard et al., 2014; Amirpourabasi et al., 2026).

In accordance with the hypothesized dysfunction of the visuo-motor integration and finger stability, the following hypotheses were set to be tested: (1) the ability to maintain a constant target force would be impaired in the MCI group compared to the HC group; (2) force generation regularity would be higher (i.e., lower adaptability) in the MCI group than in the HC group; and (3) the stability of five-finger coordination would be higher in the HC group compared to MCI group. Furthermore, as a secondary objective, we aimed to investigate the influence of target force level on these impairments, hypothesizing that (4) the disparity in force control capability between the MCI and HC groups would become more pronounced as the target force increases.

## 2 Materials and methods

### 2.1 Participants

All participants were recruited from a senior welfare center in Seoul, Republic of Korea. To assign the participants to distinct groups, we administered Korean version of the Montreal Cognitive Assessment (K-MoCA) and Korean version of the Dementia Detection Test (K-DemTect) which are widely used to diagnose MCI. Twenty-four participants were assigned to MCI group (2 male, 22 female; age: 80.04 ± 5.50) based on the following criteria: a score below 25 points on the K-MoCA, a score below 12 points on the K-DemTect, and a formal clinical diagnosis by a clinician. Twenty-four participants (2 male, 22 female; age: 75.96 ± 4.53) were recruited for the HC group (> 25 points on K-MoCA; > 12 points on K-DemTect), consisting of a similar gender distribution to the MCI group, though the average chronological age was slightly but significantly younger. The participants in both groups were strictly right-handed and had no history of severe neurological and musculoskeletal disorders (Table 1). We conducted priori power analyses based on G*Power software (version 3.1.9.7) to determine the sample size. The calculation confirmed that a total of 48 participants were required to achieve a power of 0.90, with an alpha level of 0.05, and a medium effect size (*f* = 0.25). All participants were informed of the experimental procedures and signed a written consent form before participating in this study. The consent form included not only the experimental procedures but also potential risks following the ethical guidelines of the Institutional Review Board of Yonsei University Health System, Severance Hospital (IRB No. 4-2025-0151).

**Table 1:** Participant information.

Variables	MCI	HC	*p* value
Age (years)	80.04 ± 5.50	75.96 ± 4.53	*p* = 0.007
Sex	22 Female, 2 Male	22 Female, 2 Male	–
Education (years)	8.75 ± 5.10	12.46 ± 2.65	*p* = 0.003
Handedness	All right-handed	All right-handed	–
K-MoCA	21.58 ± 2.22	27.54 ± 1.06	*p* < 0.001
K-Demtect	10.63 ± 1.31	16.42 ± 1.59	*p* < 0.001
MVC (*N*)	112.72 ± 34.87	121.40 ± 22.06	*p = 0*.154

Values are presented as mean ± standard deviation.

### 2.2 Experimental procedures

**Figure 1:**
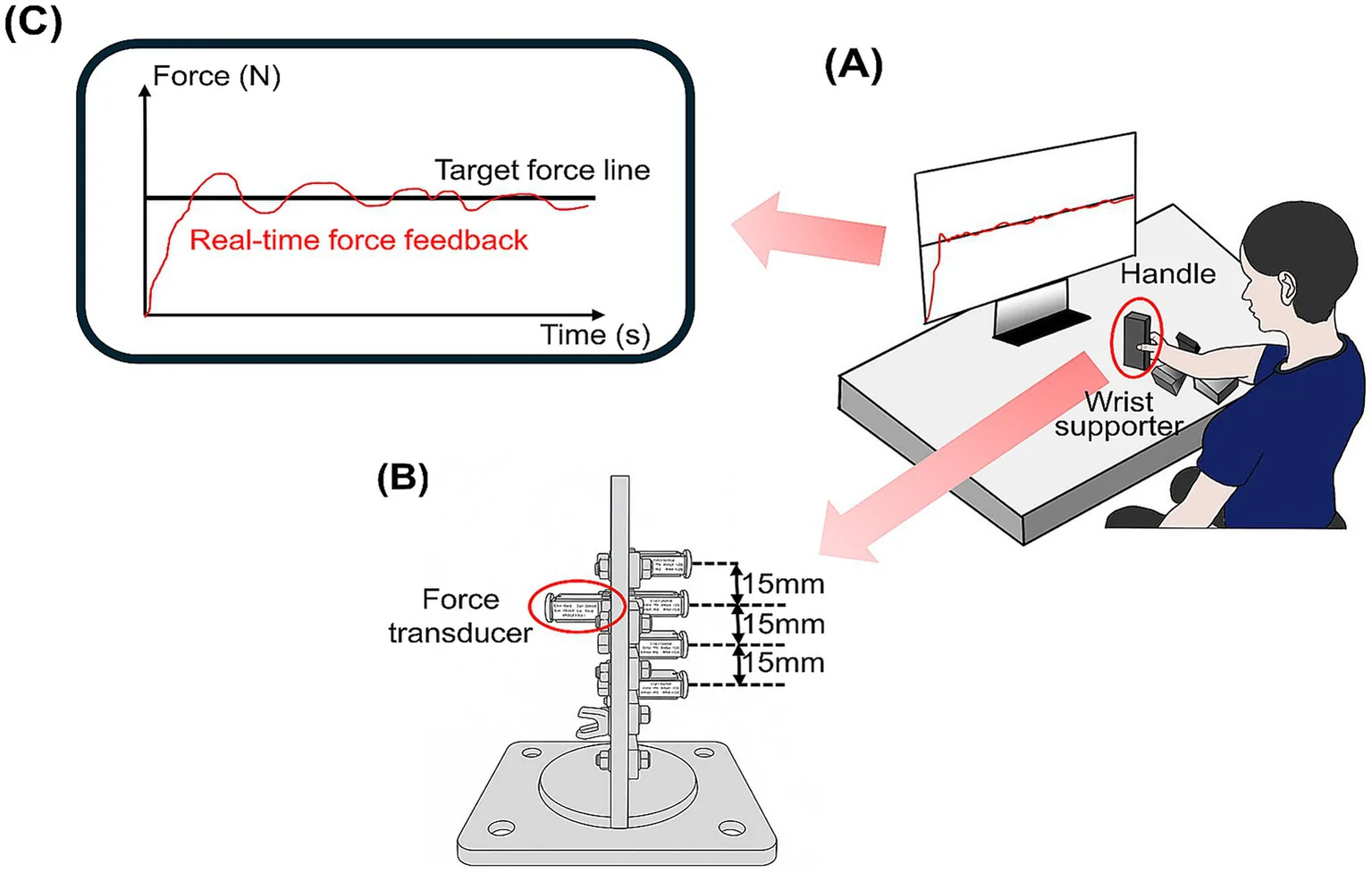
Overview of the experimental environment. **(A)** Experimental setup, **(B)** configuration of the handle, and **(C)** the visual display during the steady-state force production task.

The participants sat in a chair 80 cm away from a monitor, with their right upper arm positioned at 45° of abduction in the frontal plane, 45° of flexion in the sagittal plane, and the elbow at 30° of flexion (Figure 1A). The participants were instructed to grasp a fixed measurement device (Figure 1B), placing their fingers on each individual sensor. Five uniaxial force transducers (SSBA-S, Curiotec Co. Ltd., Korea) were attached to a customized aluminum handle to measure five individual finger forces. Considering individual hand anatomy, the placement of the transducers was adjusted for each participant. The force sensor data were sampled at 200 Hz and visual feedback of the total force of fingers was provided using a customized LabVIEW program (National Instruments, Austin, TX, United States).

#### 2.2.1 Maximal voluntary contraction force production tasks

Participants were instructed to exert maximum flexion force using all five fingers under isometric conditions and they performed two trials. For each trial, the participants were instructed to reach the maximal force within five seconds. A three-minute rest interval was provided between trials to minimize the effects of fatigue. The higher peak force of the two trials was defined as the maximal voluntary contraction (MVC). These values were subsequently utilized to calculate the target force for the following steady-state force production task.

#### 2.2.2 Steady-state force production task

Participants performed a series of steady-state force production tasks (Figure 1C), in which they were required to match the real-time force feedback (red line)—representing the sum of forces from the five fingers—to a target force line (black line). The target forces were set at 15 and 30% of their respective MVC, and each trial lasted 15 s. For each target force condition, 15 trials were conducted with a 30-s rest interval between trials to minimize neuromuscular fatigue. The order of target forces was randomized. Participants were instructed to reach the target force within 2 s and maintain it as accurately as possible for the remainder of the trial.

### 2.3 Data analysis

For the finger force data, a zero-lag, fourth-order low-pass Butterworth filter with a 10 Hz cutoff frequency was applied (Kim et al., 2018). To eliminate edge effects, the first 3 s and the last 2 s of each trial were excluded from further analysis. We calculated four types of dependent variables for each trial: (a) performance index, (b) variability of five-dimensional finger forces, (c) force regularity, and (d) dynamic stability. The mean values across the fifteen trials were used for statistical comparisons.

#### 2.3.1 Performance index during steady-state force production task

To assess the performance of steady-state force production, two outcome measures on the total force of five fingers were calculated: (a) Accuracy: root mean square error (RMSE) normalized by MVC of each participant, (b) Variability: coefficient of variation (CV; standard deviation of total force / mean of total force).

#### 2.3.2 Uncontrolled manifold analysis: variability of five-dimensional finger forces

Finger coordination during the tasks was quantified based on the uncontrolled manifold (UCM) approach (Latash et al., 2007; Park et al., 2012; Park et al., 2014). All data points (five-dimensional force data) in each trial were demeaned and the variance components, V_UCM_ and V_ORT_, were computed. A Jacobian matrix of [1; 1; 1; 1; 1] was employed, representing the linear relationship between total force and the individual finger forces. The basis vectors of the UCM space were defined by the null space of the Jacobian, representing the combinations of individual finger forces that do not affect the total force. Conversely, the orthogonal (ORT) space consists of force combinations that directly change the total force. The two components of variances (V_UCM_ and V_ORT_) were calculated as variance along these two sub-spaces and total variance (V_TOTAL_) was calculated as the sum of V_UCM_ and V_ORT_. The synergy index (ΔV) was computed as the relative difference between V_UCM_ and V_ORT_, normalized by the degrees of freedom of each space shown as Equation 1 (Song et al., 2023).


$$\mathrm{\Delta V}=\frac{\left(V_{\mathrm{UCM}}/(m-d))-(V_{\mathrm{ORT}}/d\right)}{(V_{\mathrm{UCM}}+V_{\mathrm{ORT}})/m}\;\;\;$$ (1)

Where *m* and *d* represent the degrees of freedom for the elemental variables and the performance variable, respectively. The synergy index was log-transformed using modified Fisher’s z-transformation, represented as ΔV_Z_.

#### 2.3.3 Sample entropy analysis: regularity of force production

Sample entropy (SampEn) was calculated from total finger force using to Equation 2 (Richman and Moorman, 2000). SampEn quantifies temporal structure of variability; higher values of SampEn indicate a less regular and more complex total force pattern, whereas lower values reflect higher regularity.


$$\text{SampEn}(x,m,r,N)=\ln[\frac{C_{m}(r)}{C_{m+1}(r)}]\;$$ (2)

Where *x* is the total force of fingers, *m* is the length of force template, *r* is the threshold of similarity, *N* is the total number of data points, and *C_m_*(*r*) indicates probability that any two force templates of length *m* are similar. Two templates are considered as “similar” if the absolute difference between all corresponding data points is less than the threshold *r*. However, self-matches are excluded from the calculation. We used a value of 2 for *m* and 0.2 × standard deviation of *x* for *r* according to previous studies (Yentes et al., 2013; Kim et al., 2020).

#### 2.3.4 Maximum Lyapunov exponent analysis: dynamic stability of five-dimensional finger forces

Maximum Lyapunov exponent (MLE) was calculated using Rosenstein’s algorithm (Rosenstein et al., 1993), which is highly optimized for estimating local divergence in biological time-series of modest lengths. Individual force data from the five fingers were used as the dimensions of the state space. This approach utilizes the original state space defined by the system’s physical degrees of freedom; a method supported by previous studies when all relevant state variables are observable. The integration of multiple variables into a multivariate state space is supported by research suggesting that such configurations provide a sufficiently comprehensive representation of the system’s temporal properties and complexity to be effectively utilized for MLE analysis (Gates and Dingwell, 2009; Josinski et al., 2019). To prevent the selection of temporally correlated sequential points as nearest neighbors, a temporal separation window was strictly enforced. This window was derived by performing a Fast Fourier Transform (FFT) on each of the five individual finger force time-series to compute the magnitude-weighted mean frequency for each channel. These five frequencies were then averaged to determine the overall mean frequency, and the temporal separation window was calculated by taking the inverse of the mean frequency. Based on a comprehensive visual inspection of the divergence curves across all datasets, a fixed time window of approximately 0–300 ms was selected, as this region consistently exhibited linearity. Crucially, this identical fixed window was applied uniformly to every participant and condition to eliminate any risk of artificially inflating group differences through trial-by-trial window customization. Accordingly, higher MLE values reflect a steeper slope of the divergence curve, indicating that the system is dynamically less stable.

### 2.4 Statistics

The dependent variables included RMSE, CV, V_TOTAL_, V_UCM_, V_ORT_, ΔV_Z_, SampEn and MLE. To minimize the risk of Type I errors associated with multiple comparisons across the eight dependent variables, we designated a small set of primary outcomes *a priori*. Specifically, RMSE and CV were defined as the primary outcomes because they directly tested hypothesis 1 (performance index). The remaining six variables (SampEn, V_TOTAL_, V_UCM_, V_ORT_, ΔV_Z_, and MLE) were treated as exploratory outcomes to investigate the underlying motor coordination and stability dynamics. Prior to the main analyses, statistical assumptions were rigorously verified. Data normality and homogeneity of variance were evaluated using the Shapiro–Wilk test and Levene’s test, respectively. Potential outliers were thoroughly screened based on box-plot inspections and residual analysis, and no extreme outliers were detected. Consequently, all dependent variables satisfied the required parametric assumptions. Two-way mixed-design repeated measures (RM) ANOVAs were performed to investigate the difference between the factors including *Target force* (two levels: 15 and 30%) and *Group* (two levels: MCI and HC). If any significant interaction effects were found, Bonferroni-Corrected post-hoc tests were performed. In addition, Pearson correlation analyses were performed to examine the relationships between cognitive function (results of K-MoCA and K-DemTect) and finger force control metrics (RMSE, CV, SampEn, ΔV_Z_, and MLE). For all statistical tests, the significance level was set at *p* < 0.05 and results are presented as means ± standard errors through figures. Statistical procedures were performed using IBM SPSS Statistics 22 (IBM Corp., Armonk, NY, United States).

## 3 Results

### 3.1 Performance index

Prior to evaluating force control performance, the maximum voluntary contraction (MVC) values were compared between the two groups to eliminate potential confounding effects of physical frailty or sarcopenia. An independent *t*-test revealed no statistically significant difference in absolute MVC between the MCI group and the HC group (*p* = 0.154).

**Figure 2:**
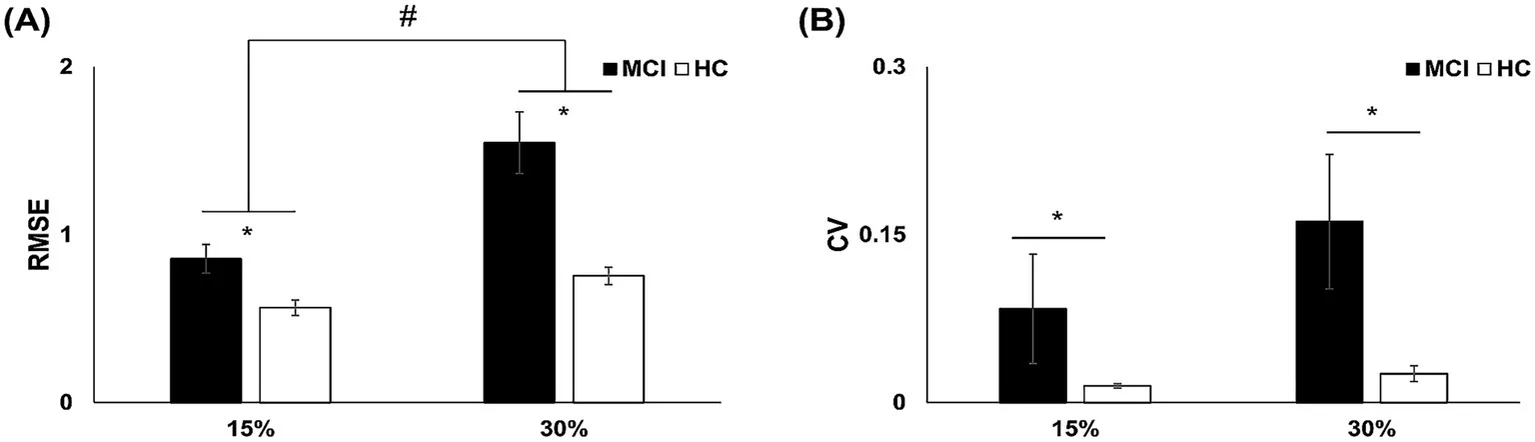
Performance index during steady-state force production task (*M* ± *SE*). **(A)** Root mean square error, and **(B)** coefficient of variation. *Asterisk* (*) denotes significant difference (*p* < 0.05) between groups. *Number sign* (#) indicates significant difference (*p* < 0.05) between target force conditions.

In general, the HC group exhibited a lower RMSE and CV compared to the MCI group, representing values that were approximately 45 and 83% lower than those of the MCI group, respectively (Figure 2). In addition, the lower target force (15%) condition resulted in lower RMSE and CV than the higher target force (30%) condition. These descriptive findings were confirmed by the subsequent statistical analyses. A significant main effect of *Group* was observed for both variables (RMSE: *F*_[1, 46]_ = 18.569, *p* < 0.001, *ηp*^2^ = 0.288; CV: *F*_[1, 46]_ = 4.146, *p* = 0.048, *ηp*^2^ = 0.083). Furthermore, the main effect of *Target force* was statistically significant only for RMSE (*F*_[1, 46]_ = 24.531, *p* < 0.001, *ηp*^2^ = 0.348), while it did not reach significance for CV. Notably, a significant Group × Target force interaction was observed for RMSE (*F*_[1, 46]_ = 7.914, *p* = 0.007, *ηp*^2^ = 0.147). Post-hoc analyses revealed that while both groups showed increased error with higher force, the MCI group demonstrated a relatively greater increase in RMSE compared to the HC group.

### 3.2 Variability of five-dimensional finger forces

**Figure 3:**
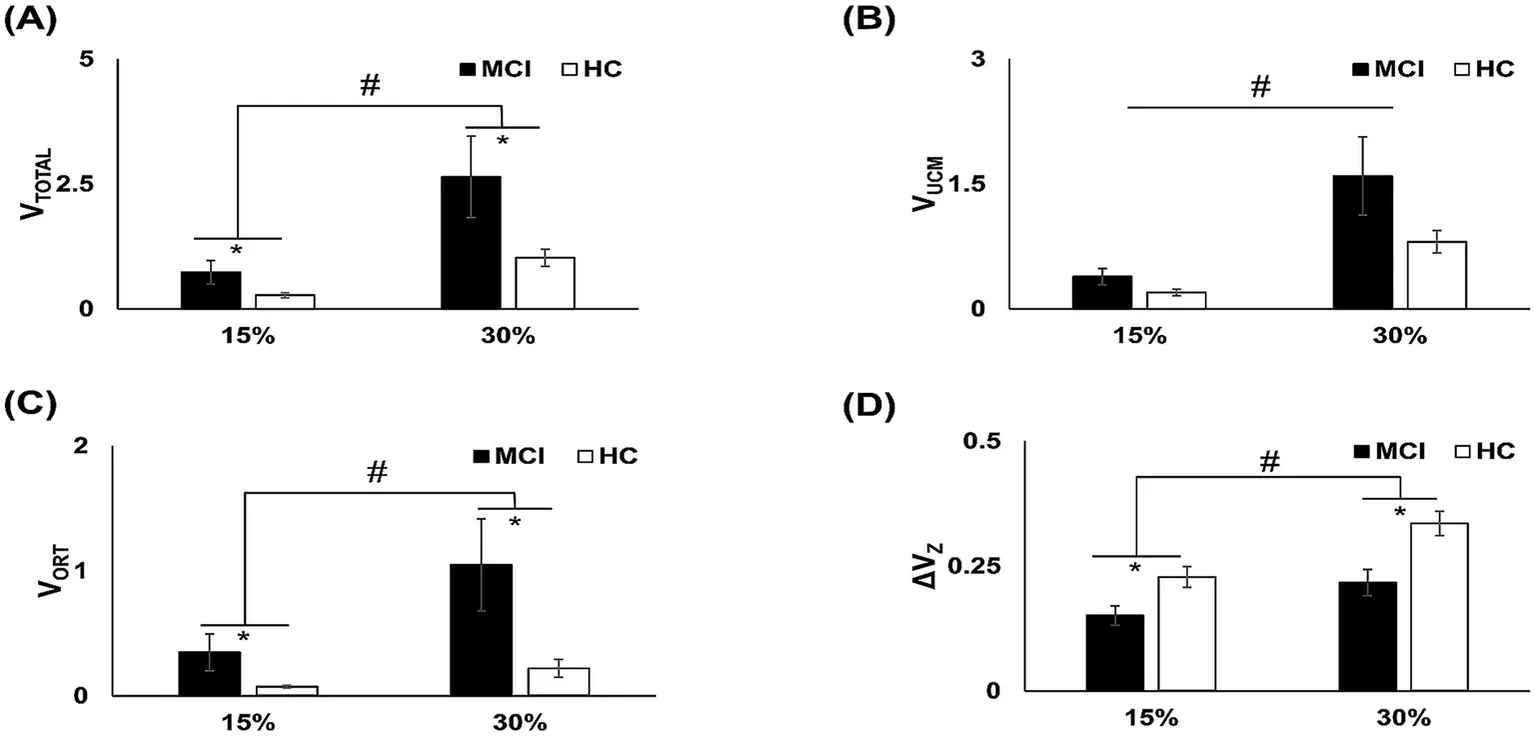
Finger force variability during steady-state force production task (*M* ± *SE*). **(A)** Variability in total dimension, **(B)** Variability in UCM space, **(C)** Variability in ORT space, and **(D)** Synergy index. *Asterisk* (*) denotes significant difference (*p* < 0.05) between groups. *Number sign* (#) indicates significant difference (*p* < 0.05) between target force conditions.

Except for ΔV_Z_, all variability components of the five-dimensional finger forces were lower in the HC group compared to the MCI group. Compared to the MCI group, the HC group exhibited reductions of approximately 61, 49, and 79% in V_TOTAL_, V_UCM_, and V_ORT_, respectively, whereas the MCI group showed a 35% lower ΔV_Z_ than the HC group (Figure 3). These results were confirmed by significant main effect of *Group* (V_TOTAL_: *F*_[1, 46]_ = 4.659, *p* = 0.036, *ηp*^2^ = 0.092; V_ORT_: *F*_[1, 46]_ = 5.839, *p* = 0.020, *ηp*^2^ = 0.113; ΔV_Z_: *F*_[1, 46]_ = 10.833, *p* = 0.002, *ηp*^2^ = 0.191). However, the difference in V_UCM_ between the two groups did not reach statistical significance. Additionally, all variability components showed similar trend and the main effect of *Target force* was statistically significant for all components; specifically, the lower target force condition showed lower values compared to the higher target force condition (V_TOTAL_: *F*_[1, 46]_ = 11.039, *p* = 0.002, *ηp*^2^ = 0.194; V_UCM_: *F*_[1, 46]_ = 15.117, *p* < 0.001, *ηp*^2^ = 0.247; V_ORT_: *F*_[1, 46]_ = 5.482, *p* = 0.024, *ηp*^2^ = 0.106; ΔV_Z_: *F*_[1, 46]_ = 35.090, *p* < 0.001, *ηp*^2^ = 0.433).

### 3.3 Regularity of force production

**Figure 4:**
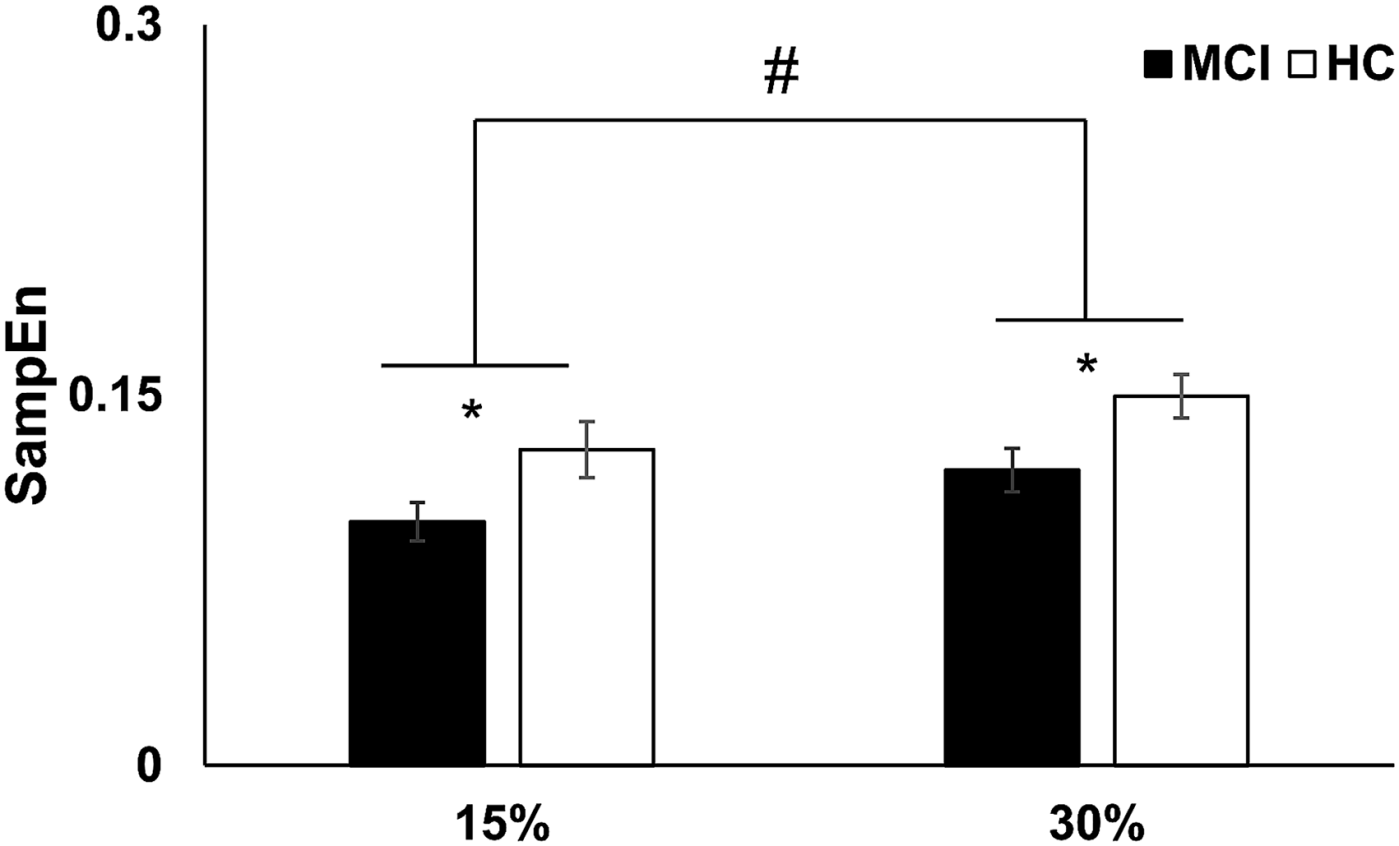
Sample entropy during steady-state force production task (*M* ± *SE*). *Asterisk* (*) denotes significant difference (*p* < 0.05) between groups. *Number sign* (#) indicates significant difference (*p* < 0.05) between target force conditions.

Overall, lower target force condition and MCI groups showed lower SampEn which means higher regularity. Specifically, the MCI group exhibited approximately 21% lower SampEn values (higher regularity) than HC group, while the lower target force condition showed an approximately 16% reduction compared to the higher target force condition. These trends were confirmed by a significant main effect of *Group* and *Target force* (*Group*: *F*_[1, 46]_ = 5.630, *p* = 0.022, *ηp*^2^ = 0.109; *Target force*: *F*_[1, 46]_ = 19.139, *p* < 0.001, *ηp*^2^ = 0.294; Figure 4).

### 3.4 Dynamic stability of five-dimensional finger forces

**Figure 5:**
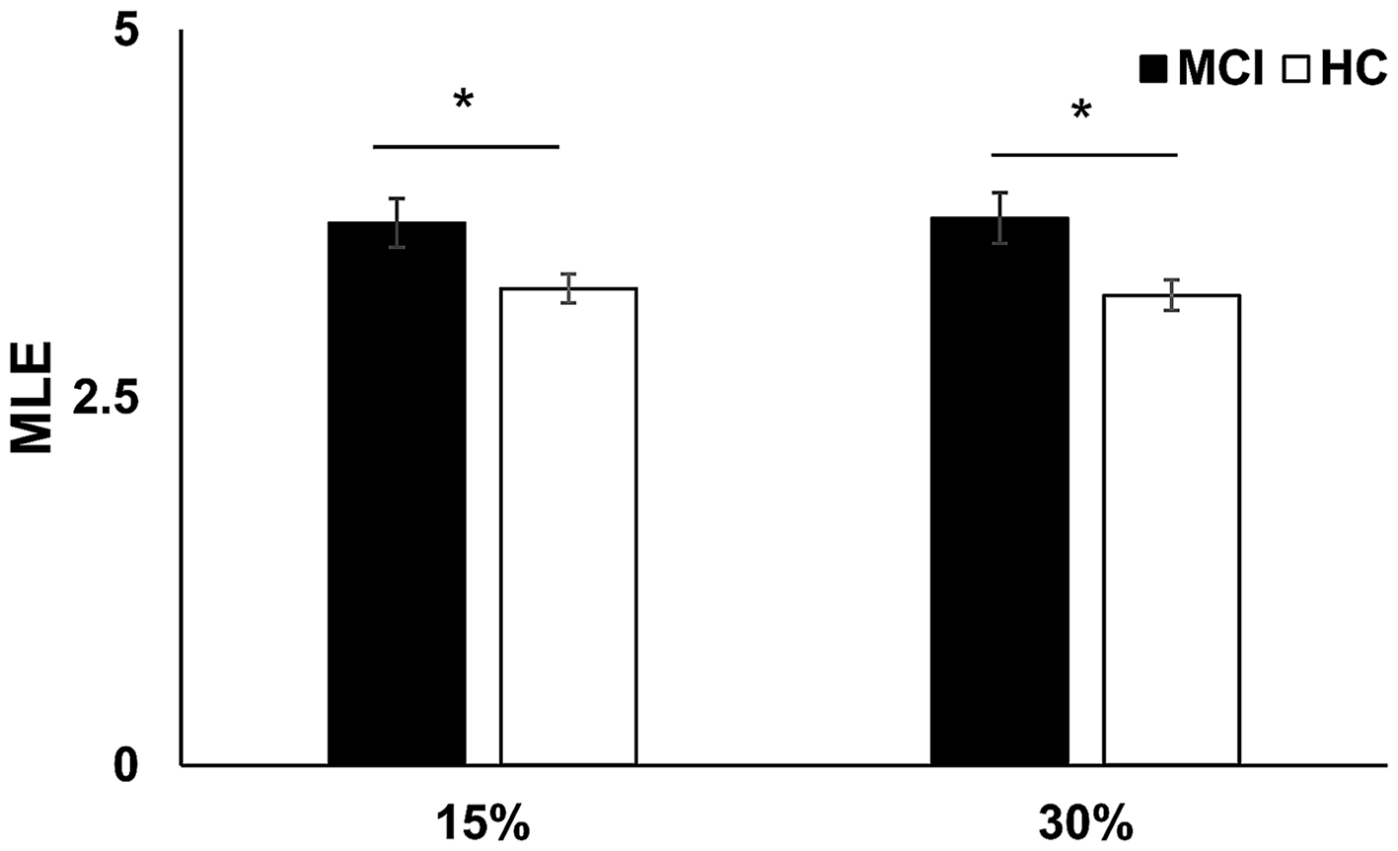
Maximum Lyapunov exponent during steady-state force production task (*M* ± *SE*). *Asterisk* (*) denotes significant difference (*p* < 0.05) between groups.

The HC group showed lower MLE compared to the MCI group, with approximate 14% discrepancy. However, there is no difference between *Target force* conditions. These observations were supported by significant main effect of *Group* (*F*_[1, 46]_ = 7.248, *p* = 0.010, *ηp*^2^ = 0.136; Figure 5).

### 3.5 Correlation between cognitive function and finger force control metrics

To investigate the relationship between cognitive status and motor performance, data from both the MCI and HC groups were pooled for a supplementary correlation analysis. When examining the relationships at each target force level, the cognitive screening scores, specifically from the K-MoCA and K-DemTect, demonstrated statistically significant correlations with the majority of the finger force control metrics (including RMSE, CV, SampEn, ΔV_Z_, and MLE). At the lower target force level, the K-MoCA score exhibited a stronger relationship with the finger force control variables (RMSE: *r* = −0.414, *p* = 0.003; CV: *r* = −0.337, *p* = 0.019; SampEn: *r* = 0.688, *p* < 0.001; ΔV_Z_: *r* = 0.293, *p* = 0.044; MLE: *r* = −0.298, *p* = 0.040) than the K-DemTect score (RMSE: *r* = −0.303, *p* = 0.036; SampEn: *r* = 0.727, *p* < 0.001; ΔV_Z_: *r* = 0.287, *p* = 0.048). A similar trend was observed in the higher target force condition, where the K-MoCA score displayed a more pronounced association (RMSE: *r* = −0.488, *p* < 0.001; CV: *r* = −0.414, *p* = 0.003; SampEn: *r* = 0.313, *p* = 0.030; ΔV_Z_: *r* = 0.438, *p* = 0.002; MLE: *r* = −0.379, *p* = 0.008) with motor performance compared to the K-DemTect score (RMSE: *r* = −0.426, *p* = 0.003; ΔV_Z_: *r* = 0.349, *p* = 0.015; MLE: *r* = −0.294, *p* = 0.042).

## 4 Discussion

The findings of the present study provide substantial support for the primary hypotheses (1, 2, and 3). According to the results, the MCI group showed lower force control capability (less ability to maintain a constant target force, higher force regularity, and lower stability of five-finger force generation) than the HC group. Regarding the secondary objective (hypothesis 4), the predicted interaction between group and target force level was significant only for the accuracy index (RMSE) and not for the other metrics. In the subsequent sections, we will examine these characteristics of individuals with MCI, focusing on both total force level and individual force level of five-finger force production.

### 4.1 Capability of generating steady-state submaximal force with visual feedback: net force level

The task required participants to maintain a target force as accurately as possible using visual feedback. Therefore, RMSE and CV serve as critical indicators of task performance. Our results demonstrate that the MCI group exhibited significantly higher RMSE and CV compared to the HC group, suggesting a diminished capability to generate and sustain a constant target force. Furthermore, a significant interaction between group and target force level was observed in RMSE, indicating that the increase in RMSE at higher force levels was more pronounced in the MCI group than in the HC group. These findings indicate that the visuo-motor integration task utilized in the present study can effectively capture functional difference between healthy controls and individuals with MCI. Given that controlling higher target forces requires greater PFC activation (Mandrick et al., 2013), the finding that the MCI group exhibited lower accuracy than the control group as target force increased suggests that their impaired motor control may be related to the altered PFC dependent neural regulation. However, as no direct neural measures (e.g., EEG, fNIRS) were obtained in the present study, this remains a speculative interpretation to be tested in future research incorporating neurophysiological or neuroimaging techniques.

Additionally, SampEn, an indicator of force generation regularity, revealed that the MCI group exhibited a more regular (less complex) force pattern. Given that the time interval (10–15 ms) utilized for this analysis is significantly shorter than the typical latency required for spinal reflexes or voluntary motor corrections, one possible interpretation is that these alterations reflect subclinical adjustments within peripheral or spinal motor structures, rather than solely central mechanisms. Increased motor-unit synchronization—which would reduce the asynchronous firing that normally supports complex, adaptable force output (Madarshahian et al., 2021) – represents one candidate mechanism that could plausibly contribute to the more rigid and regular force patterns observed in the MCI group (Semmler, 2002). However, because electromyographical (EMG) signals were not directly recorded in the present study, this motor-unit synchronization framework remains a speculative physiological hypothesis rather than a direct finding.

### 4.2 Characteristics of multi-finger coordination: individual finger force level

One of the primary strengths of the present study lies in its utilization of a multi-finger force production task, which provides a sensitive window into the neural control of movement and the functional integrity of the brain. By analyzing five-dimensional force data, we were able to capture the complex, high dimensional nature (coordination pattern among fingers) of the motor control system in the MCI group.

The results of the current study indicate that the MCI group exhibited greater total variability in five-finger force production compared to the HC group. However, increased variability is not inherently negative. In the context of the UCM framework, variance among individual fingers can be interpreted as evidence of synergistic compensation, where fingers co-vary to stabilize a performance (Reschechtko et al., 2017; Kong et al., 2019). Notably, the group difference between the MCI and HC groups was more pronounced in the ORT space than in the UCM space. These findings suggest that individuals with MCI tend to employ finger force combinations that promote fluctuations in the net force rather than those that stabilize the target force. This can be understood as a diminished ability to suppress task-irrelevant noise, leading to a failure in maintaining a consistent total force. One possible interpretation is that this tendency relates to structural or functional changes within the basal ganglia, a region previously implicated as a key neural structure governing the synergistic control of multi-finger forces (Park et al., 2014). However, in the absence of direct neuroimaging data in the present study, this should be regarded as a candidate mechanism for future investigation rather than a conclusion supported by the current findings.

While the UCM analysis focuses on the spatial organization of finger forces throughout the task, the MLE analysis provides insight into the temporal structure and stability of those force outputs. Our findings revealed that the MCI group exhibited higher MLE values compared with the HC group, indicating that their force production patterns were less stable and diverged more rapidly over time. Together, these results demonstrate that motor control deficits in individuals with MCI are reflected not only in the overall magnitude of variance but also in the underlying time-series dynamics, where the system fails to maintain a stable trajectory of multi-finger coordination.

## 5 Limitations

One of the primary limitations of this study is that participants were recruited as a general MCI group without distinguishing clinical subtypes (e.g., amnestic vs. non-amnestic), due to the lack of a detailed neuropsychological battery at screening. Because motor deficits may manifest differently across MCI subtypes, this heterogeneity constrains our neural interpretation: a predominantly non-amnestic profile would support our prefrontal/executive-motor framing, whereas an amnestic-weighted cohort would favor a hippocampal/medial temporal interpretation. As subgroup composition could not be systematically determined, our neural interpretation remains exploratory, and future studies should recruit well-characterized MCI subtypes (e.g., non-amnestic cohorts) to test these mechanisms more directly. In addition, our sample showed a marked sex imbalance (22 women, 2 men per group). Although the two male participants’ data fell within the female distribution without appearing as outliers, MVC normalization cannot fully account for sex-specific differences in neuromotor aging, and generalization to male or mixed-sex populations should be made cautiously. Future studies should stratify participants by subtype and sex to investigate these specific characteristics more comprehensively.

The two groups significantly differed in age and education. To address this, separate sensitivity analyses (ANCOVA) were conducted, entering age and education as covariates individually, with full results of between-group differences presented in the Supplementary material.

Lastly, the cross-sectional design further limits our ability to establish how coordination deficits relate to the progression of cognitive decline; although secondary correlations linked screening scores to force control metrics, longitudinal follow-up is needed to capture how these measures evolve with cognitive stage over time.

## 6 Conclusion

However, the present study distinguishes itself by employing a multi-finger coordination paradigm, revealing that individuals with MCI exhibit multifaceted deficits in the motor control system. These deficits are specifically characterized by: (1) deteriorated visuo-motor integration, (2) rigid and less adaptable force patterns, and (3) increased spatial and temporal instability in multi-finger coordination. These findings underscore that the coordination among multiple fingers provides a valuable window into the intrinsic neuromotor characteristics of the motor control system. Ultimately, this study offers a potential framework of the multi-finger coordination task as objective neuromechanical marker for MCI screening.

## Data Availability

The raw data supporting the conclusions of this article will be made available by the authors, without undue reservation.

## References

[ref1] Amirpourabasi A. Lamb S. E. Chow J. Y. Williams G. K. R. (2026). Using nonlinear dynamic analysis to differentiate fall status in older women. Gait Posture 124:110032. doi: 10.1016/j.gaitpost.2025.110032, 41202477

[ref2] Beauchet O. Launay C. P. Annweiler C. Allali G. (2015). Hippocampal volume, early cognitive decline and gait variability: which association? Exp. Gerontol. 61, 98–104. doi: 10.1016/j.exger.2014.11.002 25446977

[ref3] Binkofski F. Buccino G. Stephan K. M. Rizzolatti G. Seitz R. J. Freund H. J. (1999). A parieto-premotor network for object manipulation: evidence from neuroimaging. Exp. Brain Res. 128, 210–213. doi: 10.1007/s002210050838, 10473761

[ref4] Boripuntakul S. Kamnardsiri T. Lord S. R. Maiarin S. Worakul P. Sungkarat S. (2022). Gait variability during abrupt slow and fast speed transitions in older adults with mild cognitive impairment. PLoS One 17:e0276658. doi: 10.1371/journal.pone.0276658, 36269750 PMC9586342

[ref5] Gates D. H. Dingwell J. B. (2009). Comparison of different state space definitions for local dynamic stability analyses. J. Biomech. 42, 1345–1349. doi: 10.1016/j.jbiomech.2009.03.015, 19380140 PMC2718682

[ref6] Han Y. Lui S. Kuang W. Lang Q. Zou L. Jia J. (2012). Anatomical and functional deficits in patients with amnestic mild cognitive impairment. PLoS One 7:e28664. doi: 10.1371/journal.pone.0028664, 22319555 PMC3272002

[ref7] Josinski H. Switonski A. Michalczuk A. Grabiec P. Pawlyta M. Wojciechowski K. (2019). Assessment of local dynamic stability in gait based on univariate and multivariate time series. Comput. Math. Methods Med. 2019:6917658. doi: 10.1155/2019/6917658 31428185 PMC6683834

[ref8] Kim H. J. Kang N. Cauraugh J. H. (2020). Transient changes in paretic and non-paretic isometric force control during bimanual submaximal and maximal contractions. J. Neuroeng. Rehabil. 17:64. doi: 10.1186/s12984-020-00693-3, 32410626 PMC7227276

[ref9] Kim K. Xu D. Park J. (2018). Effect of kinetic degrees of freedom on multi-finger synergies and task performance during force production and release tasks. Sci. Rep. 8:12758. doi: 10.1038/s41598-018-31136-8, 30143688 PMC6109105

[ref10] Kluger A. Gianutsos J. G. Golomb J. Ferris S. H. George A. E. Franssen E. . (1997). Patterns of motor impairement in normal aging, mild cognitive decline, and early Alzheimer's disease. J. Gerontol. B Psychol. Sci. Soc. Sci. 52B, P28–P39. doi: 10.1093/geronb/52B.1.P28 9008673

[ref11] Kong J. Kim K. Joung H. J. Chung C. Y. Park J. (2019). Effects of spastic cerebral palsy on multi-finger coordination during isometric force production tasks. Exp. Brain Res. 237, 3281–3295. doi: 10.1007/s00221-019-05671-3, 31664488

[ref12] Latash M. L. Scholz J. P. Schoner G. (2007). Toward a new theory of motor synergies. Mot. Control. 11, 276–308. doi: 10.1123/mcj.11.3.276, 17715460

[ref13] Madarshahian S. Letizi J. Latash M. L. (2021). Synergic control of a single muscle: the example of flexor digitorum superficialis. J. Physiol. 599, 1261–1279. doi: 10.1113/jp280555, 33206377

[ref14] Madsen S. K. Ho A. J. Hua X. Saharan P. S. Toga A. W. Jack C. R. JR. . (2010). 3D maps localize caudate nucleus atrophy in 400 Alzheimer's disease, mild cognitive impairment, and healthy elderly subjects. Neurobiol. Aging 31, 1312–1325. doi: 10.1016/j.neurobiolaging.2010.05.002 20538376 PMC2903198

[ref15] Malek-Ahmadi M. Powell J. J. Belden C. M. O'connor K. Evans L. Coon D. W. . (2015). Age- and education-adjusted normative data for the Montreal cognitive assessment (MoCA) in older adults age 70-99. Neuropsychol. Dev. Cogn. B Aging Neuropsychol. Cogn. 22, 755–761. doi: 10.1080/13825585.2015.1041449 25942388

[ref16] Mandrick K. Derosiere G. Dray G. Coulon D. Micallef J. P. Perrey S. (2013). Prefrontal cortex activity during motor tasks with additional mental load requiring attentional demand: a near-infrared spectroscopy study. Neurosci. Res. 76, 156–162. doi: 10.1016/j.neures.2013.04.006, 23665138

[ref17] Mao C. Mo Y. Jiang J. Fang S. Hu Z. Ke Z. . (2025). Association between high plasma p-tau181 level and gait changes in patients with mild cognitive impairment. Sci. Rep. 15:14679. doi: 10.1038/s41598-025-94472-6, 40287471 PMC12033327

[ref18] Moro S. B. Carrieri M. Avola D. Brigadoi S. Lancia S. Petracca A. . (2016). A novel semi-immersive virtual reality visuo-motor task activates ventrolateral prefrontal cortex: a functional near-infrared spectroscopy study. J. Neural Eng. 13:036002. doi: 10.1088/1741-2560/13/3/036002, 27001948

[ref19] Pa J. Boxer A. Chao L. L. Gazzaley A. Freeman K. Kramer J. . (2009). Clinical-neuroimaging characteristics of dysexecutive mild cognitive impairment. Ann. Neurol. 65, 414–423. doi: 10.1002/ana.21591, 19399879 PMC2680500

[ref20] Park J. Lewis M. M. Huang X. Latash M. L. (2014). Dopaminergic modulation of motor coordination in Parkinson's disease. Parkinsonism Relat. Disord. 20, 64–68. doi: 10.1016/j.parkreldis.2013.09.019 24090949 PMC3946854

[ref21] Park J. Wu Y. H. Lewis M. M. Huang X. Latash M. L. (2012). Changes in multifinger interaction and coordination in Parkinson's disease. J. Neurophysiol. 108, 915–924. doi: 10.1152/jn.00043.2012, 22552184 PMC3424084

[ref22] Petersen R. C. Doody R. Kurz A. Mohs R. C. Morris J. C. Rabins P. V. . (2001). Current concepts in mild cognitive impairment. Arch. Neurol. 58, 1985–1992. doi: 10.1001/archneur.58.12.1985, 11735772

[ref23] Reschechtko S. Zatsiorsky V. M. Latash M. L. (2017). The synergic control of multi-finger force production: stability of explicit and implicit task components. Exp. Brain Res. 235, 1–14. doi: 10.1007/s00221-016-4768-4, 27601252 PMC5226884

[ref24] Reynard F. Vuadens P. Deriaz O. Terrier P. (2014). Could local dynamic stability serve as an early predictor of falls in patients with moderate neurological gait disorders? A reliability and comparison study in healthy individuals and in patients with paresis of the lower extremities. PLoS One 9:e100550. doi: 10.1371/journal.pone.0100550, 24949737 PMC4065053

[ref25] Richman J. S. Moorman J. R. (2000). Physiological time-series analysis using approximate entropy and sample entropy. Am. J. Physiol. Heart Circ. Physiol. 278, H2039–H2049. doi: 10.1152/ajpheart.2000.278.6.H2039, 10843903

[ref26] Roalf D. R. Rupert P. Mechanic-Hamilton D. Brennan L. Duda J. E. Weintraub D. . (2018). Quantitative assessment of finger tapping characteristics in mild cognitive impairment, Alzheimer's disease, and Parkinson's disease. J. Neurol. 265, 1365–1375. doi: 10.1007/s00415-018-8841-8, 29619565 PMC5992087

[ref27] Rosenstein M. T. Collins J. J. De Luca C. J. (1993). A practical method for calculating largest Lyapunov exponents from small data sets. Phys. D 65, 117–134. doi: 10.1016/0167-2789(93)90009-p

[ref28] Rossetti H. C. Lacritz L. H. Cullum C. M. Weiner M. F. (2011). Normative data for the Montreal cognitive assessment (MoCA) in a population-based sample. Neurology 77, 1272–1275. doi: 10.1212/wnl.0b013e318230208a, 21917776

[ref29] Semmler J. G. (2002). Motor unit synchronization and neuromuscular performance. Exerc. Sport Sci. Rev. 30, 8–14. 11800501 10.1097/00003677-200201000-00003

[ref30] Song J. Kim K. Park J. (2023). Multi-muscle synergies of postural control in self- and external-triggered force release during simulated archery shooting. J. Mot. Behav. 55, 289–301. doi: 10.1080/00222895.2023.2187336, 36919981

[ref31] Stergiou N. Decker L. M. (2011). Human movement variability, nonlinear dynamics, and pathology: is there a connection? Hum. Mov. Sci. 30, 869–888. doi: 10.1016/j.humov.2011.06.002, 21802756 PMC3183280

[ref32] Strongman C. Morrison A. (2020). A scoping review of non-linear analysis approaches measuring variability in gait due to lower body injury or dysfunction. Hum. Mov. Sci. 69:102562. doi: 10.1016/j.humov.2019.102562, 31989953

[ref33] Tabatabaei-Jafari H. Shaw M. E. Cherbuin N. (2015). Cerebral atrophy in mild cognitive impairment: a systematic review with meta-analysis. Alzheimers Dement. (Amst.) 1, 487–504. doi: 10.1016/j.dadm.2015.11.002 27239527 PMC4879488

[ref34] Teipel S. J. Born C. Ewers M. Bokde A. L. Reiser M. F. Moller H. J. . (2007). Multivariate deformation-based analysis of brain atrophy to predict Alzheimer's disease in mild cognitive impairment. NeuroImage 38, 13–24. doi: 10.1016/j.neuroimage.2007.07.008, 17827035

[ref35] VAN Emmerik R. E. A. Ducharme S. W. Amado A. C. Hamill J. (2016). Comparing dynamical systems concepts and techniques for biomechanical analysis. J. Sport Health Sci. 5, 3–13. doi: 10.1016/j.jshs.2016.01.013, 30356938 PMC6191988

[ref36] Yentes J. M. Hunt N. Schmid K. K. Kaipust J. P. Mcgrath D. Stergiou N. (2013). The appropriate use of approximate entropy and sample entropy with short data sets. Ann. Biomed. Eng. 41, 349–365. doi: 10.1007/s10439-012-0668-3, 23064819 PMC6549512

