# Supplementary material for: Altered regularity and stability of multi-finger force production in individuals with mild cognitive impairment
Source: Front Hum Neurosci. 2026 Sep 1;20:1873097. doi: 10.3389/fnhum.2026.1873097 (PMC13574785; doi:10.3389/fnhum.2026.1873097)
Supplement: Supplementary file 1 [file Table_1.docx]

ANCOVA results confirmed that our findings were not confounded by education level. However, while primary outcomes remained robust after controlling for age, secondary indices such as SampEn and MLE were sensitive to age, and these results should therefore be interpreted with caution.

| **Supplementary table.** Results of ANCOVA on between-group differences | | | | |
| --- | --- | --- | --- | --- |
| Covariate | Variable | *F* value | *p* value | Effect size (*ηp*^2^) |
| Age | RMSE | *F*_[1,45]_ = 14.550 | *p* < .001 | 0.244 |
|  | CV | *F*_[1,45]_ = 5.230 | *p* = .027 | 0.104 |
|  | V_TOTAL_ | *F*_[1,45]_ = 5.538 | *p* = .014 | 0.127 |
|  | V_UCM_ | *F*_[1,45]_ = 4.841 | *p* = .033 | 0.097 |
|  | V_ORT_ | *F*_[1,45]_ = 7.429 | *p* = .009 | 0.142 |
|  | ΔV_Z_ | *F*_[1,45]_ = 4.468 | *p* = .040 | 0.090 |
|  | SampEn | *F*_[1,45]_ = 3.079 | *p* = .086 | 0.064 |
|  | MLE | *F*_[1,45]_ = 3.477 | *p* = .069 | 0.072 |
| Education | RMSE | *F*_[1,45]_ = 17.474 | *p* < .001 | 0.280 |
|  | CV | *F*_[1,45]_ = 4.191 | *p* = .046 | 0.085 |
|  | V_TOTAL_ | *F*_[1,45]_ = 6.729 | *p* = .013 | 0.130 |
|  | V_UCM_ | *F*_[1,45]_ = 5.065 | *p* = .029 | 0.101 |
|  | V_ORT_ | *F*_[1,45]_ = 7.522 | *p* = .009 | 0.143 |
|  | ΔV_Z_ | *F*_[1,45]_ = 8.429 | *p* = .006 | 0.158 |
|  | SampEn | *F*_[1,45]_ = 6.465 | *p* = .015 | 0.126 |
|  | MLE | *F*_[1,45]_ = 7.383 | *p* = .009 | 0.141 |
|  | | | | |
